# Acceptance of Vaccinations in Pandemic Outbreaks: A Discrete Choice Experiment

**DOI:** 10.1371/journal.pone.0102505

**Published:** 2014-07-24

**Authors:** Domino Determann, Ida J. Korfage, Mattijs S. Lambooij, Michiel Bliemer, Jan Hendrik Richardus, Ewout W. Steyerberg, Esther W. de Bekker-Grob

**Affiliations:** 1 Department of Public Health, Erasmus MC, University Medical Center Rotterdam, Rotterdam, The Netherlands; 2 Center for Prevention and Health Services Research, National Institute for Public Health and the Environment (RIVM), Bilthoven, The Netherlands; 3 Institute of Transport and Logistics Studies, The University of Sydney Business School, Sydney, New South Wales, Australia; 4 Rotterdam-Rijnmond Public Health Service, Rotterdam, The Netherlands; University of Nebraska-Lincoln, United States of America

## Abstract

**Background:**

Preventive measures are essential to limit the spread of new viruses; their uptake is key to their success. However, the vaccination uptake in pandemic outbreaks is often low. We aim to elicit how disease and vaccination characteristics determine preferences of the general public for new pandemic vaccinations.

**Methods:**

In an internet-based discrete choice experiment (DCE) a representative sample of 536 participants (49% participation rate) from the Dutch population was asked for their preference for vaccination programs in hypothetical communicable disease outbreaks. We used scenarios based on two disease characteristics (susceptibility to and severity of the disease) and five vaccination program characteristics (effectiveness, safety, advice regarding vaccination, media attention, and out-of-pocket costs). The DCE design was based on a literature review, expert interviews and focus group discussions. A panel latent class logit model was used to estimate which trade-offs individuals were willing to make.

**Results:**

All above mentioned characteristics proved to influence respondents’ preferences for vaccination. Preference heterogeneity was substantial. Females who stated that they were never in favor of vaccination made different trade-offs than males who stated that they were (possibly) willing to get vaccinated. As expected, respondents preferred and were willing to pay more for more effective vaccines, especially if the outbreak was more serious (€6–€39 for a 10% more effective vaccine). Changes in effectiveness, out-of-pocket costs and in the body that advises the vaccine all substantially influenced the predicted uptake.

**Conclusions:**

We conclude that various disease and vaccination program characteristics influence respondents’ preferences for pandemic vaccination programs. Agencies responsible for preventive measures during pandemics can use the knowledge that out-of-pocket costs and the way advice is given affect vaccination uptake to improve their plans for future pandemic outbreaks. The preference heterogeneity shows that information regarding vaccination needs to be targeted differently depending on gender and willingness to get vaccinated.

## Introduction

Worldwide viral infection outbreaks with, e.g. Influenza A(H1N1), SARS and H5N1 avian influenza, have been of serious impact in the past [1]. If a new outbreak would occur, the global spread is likely to be very rapid due to increased travel and urbanization [2]. Extrapolation of the 1918–1920 avian influenza pandemic mortality rates indicates that 62 million people would be killed if a similar pandemic would happen these days [3]. Preventive measures, such as social distancing measures or vaccination programs, are very important in limiting the spread of new viruses [4], [5]. However, the lack of willingness to act according to such measures in crisis situations has proven to be a major issue in the European Union [6]. Consequently, it is important to have insights into what motivates individual people to decide for or against vaccination. If motivations are known, these can be addressed in pandemic preparedness plans and vaccination strategies to increase vaccination rates and thus reduce the spread of viral outbreaks. Furthermore, insight in motivations can lead to an accurate prediction of the uptake of vaccinations, which is helpful when implementing vaccination programs.

Various studies have been conducted to explore reasons why individual members of the general public accepted or declined pandemic vaccinations, especially focusing on the Influenza A(H1N1) pandemic of 2009 [6]–[8]. These studies showed that participation in vaccination programs is based on weighing the burden of the vaccination (e.g. risk of side effects), against its potential benefits (e.g. reduce the risk of infection), in a given context (e.g. severity of first cases of the disease). Several European countries reported that public perception factors, such as poor confidence in the need for the vaccine and concerns about the relatively new vaccine, may have contributed to the low vaccination coverage rates during the Influenza A (H1N1) pandemic of 2009 [9]. Despite the presence of studies investigating reasons of members of the general public to get vaccinated or not, quantitative studies that assess the relative importance of these reasons are lacking. It is precisely this information that is needed to make highly effective health care policy plans regarding pandemic outbreaks and vaccinations.

The aim of this study is to investigate the preferences of the general population for pandemic vaccinations quantitatively. Additionally, we aim to calculate the expected uptake of base case vaccination programs for certain hypothetical outbreaks. The current study is conducted within the scope of the project Effective Communication in Outbreak Management: development of an evidence-based tool for Europe (E-com@eu, http://www.ecomeu.info/). This project aims to develop an evidence-based behavioral and communication strategy for health professionals and agencies throughout Europe in case of major outbreaks, by integration of social, behavioral, communication, and media sciences.

## Methods

### Ethics Statement

A declaration of no objection was received from the Medical Ethics Committee of the Erasmus MC, University Medical Center Rotterdam (MEC-2012-263) after they reviewed the study protocol. The methodology of this study, a survey amongst healthy volunteers of an internet panel, does not fall within the scope of the Medical Research Involving Human Subjects Act (in Dutch: WMO). Although the aim of the study is of medical nature, participants are not being subjected to any treatment or behavioral adjustments.

### Discrete choice experiments

DCE methodology is a survey-based stated preference technique to quantitatively investigate individual preferences. DCEs have been widely used in health care to examine stakeholder preferences [10], [11] and have been previously used to examine preferences for non-emergency vaccination programs, such as Human Papilloma Virus (HPV) vaccinations and seasonal influenza vaccinations [12], [13]. In DCEs, it is assumed that a medical intervention, such as a vaccination program, can be described by its characteristics (attributes; e.g. effectiveness of a vaccine, safety of the vaccine, and costs of the vaccine). Those characteristics are further specified by variants of that characteristic (attribute levels; e.g. for effectiveness of a vaccine: 30%, 50%, 70% and 90% effective). A second assumption is that the individual’s preference for a medical intervention is determined by the levels of those attributes [14]. The relative importance of attributes can be assessed by presenting respondents a series of questions in which they are asked to choose a preferred alternative from a set of two or more hypothetical intervention alternatives with varying combinations of attribute levels [15]. DCEs are based on Lancaster’s consumer theory [16] and random utility theory (RUT) [17] which assume that an individual acts rationally and always chooses the alternative with the highest level of utility. We followed recent guidelines for good DCE practice [18], [19].

### Selection of attributes and attribute levels

Only a limited number of attributes and attribute levels can be used in a DCE, since otherwise the precision and reliability of the results will decrease. On the other hand, one also needs to include all relevant attributes and attribute levels to avoid that respondents make significant inferences on omitted attributes or levels [19], [20].

To obtain insights into possible attributes and their levels to be included in this DCE, we conducted a strategic literature search in three databases (searching for literature related to DCEs and/or vaccination preferences in PubMed, Embase and PsychINFO), semi-structured expert interviews and a focus group study. For the expert interviews, we have spoken to nine experts of different relevant fields, e.g. infectious diseases, vaccinations, preventive behavior and implementation of preventive measures. For the focus group study, we conducted seven focus group discussions with the general population from the Netherlands; targeting urban populations (two groups); populations of more rural areas (two groups); and ethnic minorities in the Netherlands (three groups). Eligible participants were recruited by a research company and via the network of a researcher of the department of Public Health of the Erasmus MC, University Medical Center Rotterdam, using purposive sampling to ensure a diverse sample. Participants were informed that they would receive a financial incentive (40 euros) for their contribution and to cover travel costs and they were informed that the data would be analyzed anonymously. All participants gave written informed consent prior to the discussions. All focus groups were audio taped, transcribed verbatim and anonymously. The transcripts were analyzed using thematic analysis using NVivo Software (version 10, http://www.qsrinternational.com). The focus group study approach was included in the study protocol for which a declaration of no objection was received from the Medical Ethics Committee of the Erasmus MC, University Medical Center Rotterdam. We used a topic list based on the literature search and on two theoretic models, i.e. the Health Belief Model [21] and to a lesser extent the Protection Motivation Theory [22], to structure the focus group discussions on outbreaks of new diseases and preventive measures. These models assume that people react to a perceived threat, by performing some action. The level of threat depends on the perceived susceptibility to a disease and the perceived severity of a disease. People weigh this threat to perceived benefits (such as effectiveness) and barriers (such as costs) of actions. The model assumes that also other factors influence someone’s intention to take some action, such as cues to action (e.g. media attention) and variables (age, sex, peer pressure etc.). We used these models as a base for the topic list because of their largely empirically tested ability to explain and predict intention of and complying with preventive medical care recommendations, including vaccinations [7], [23], [24]. Additionally, during the focus group discussions, participants were asked to write down and rank the most important reasons for them to get vaccinated during future pandemic outbreaks.

Using these results and through extensive discussion with E-com@eu project members, we selected two disease specific scenario variables and five vaccination program attributes and their corresponding levels (Table 1). For each scenario (which is a combination of the susceptibility to the disease and severity of the disease), three alternatives were presented, namely (i) No vaccination, (ii) Vaccination A, and (iii) Vaccination B, where the latter two are represented by combinations of effectiveness, safety, advice, media and out-of-pocket costs. We aimed at selecting a sufficient wide range of attribute levels that are realistic now and will remain so in the near future and levels that were relevant to policy as well as plausible and understandable for the respondents. Furthermore, for each continuous attribute we selected at least three levels to be able to test for non-linear relationships.

**Table 1:** Scenario variables, vaccination program attributes and their levels included in the DCE survey.

Scenario variables	Levels
Susceptibility to the disease^1^	5%
	10%
	20%
Severity of the disease^2^	5%
	25%
	50%
	75%
**Vaccination program attributes**	**Levels**
Effectiveness of vaccine	30%
	50%
	70%
	90%
Safety of the vaccine^3,4^	Unknown, expected to be safe (reference level)
	Unknown, no experience with similar vaccines yet
Advice regarding the vaccine^3^	Family and/or friends recommend vaccination (reference level)
	Family and/or friends discourage vaccination
	Your doctor recommends vaccination
	Your doctor discourages vaccination
	Dutch government & RIVM recommend vaccination
	International organizations recommend vaccination
Media coverage about the vaccine^3^	Traditional media^5^ positive (reference level)
	Traditional media^5^ negative
	Social/interactive media^6^ positive
	Social/interactive media^6^ negative
Out-of-pocket costs	€0
	€50
	€100

Notes: Levels of the no vaccination option were defined as: not applicable (n.a.), no side effects, n.a., n.a., €0 respectively. The scenario variables were the same across all alternatives in one choice set. Abbreviation used: RIVM = Dutch abbreviation of National Institute for Public Health and the Environment. (1) Defined as the proportion of population infected with the new disease, i.e. having symptoms. (2) Defined as the proportion of infected population that suffered severe symptoms (death, life-threatening events, hospitalization and severe or permanent handicap). (3) The attributes ‘safety of the vaccine’, ‘advice about the vaccine’ and ‘media coverage about the vaccine’ entered the analysis as categorical variables. (4) Long term severe side effects (death, life-threatening events, hospitalization, severe or permanent handicap, or side effects leading to birth defects to an unborn fetus). Before the start of the choice tasks, respondents were informed that on the short term, vaccinations resulted in mild side effects only. (5) Traditional media were defined as: radio, newspapers and television. (6) Social/interactive media were defined as: blogs, Twitter and social network websites.

### Study design and questionnaire

If all combinations of attribute levels were to be presented in choice sets, this would have led to 576 (2^1^ * 3^1^ * 4^2^ * 6^1^) hypothetical vaccination alternatives for 12 (3^1^ * 4^1^) different disease outbreaks (scenarios). As it is not feasible to present a single individual with all these scenarios and alternatives (i.e. full factorial design), a subset of scenarios and alternatives (i.e. fractional factorial design) was generated [25]. Zero priors for all categorical variables and best-guess priors for all linear variables were used to generate an efficient design by maximizing D-efficiency (using Ngene software, version 1.1.1, http://www.choice-metrics.com/). With this design we were able to estimate all main effects and a number of two-way and higher order interactions between attributes. Presenting a single individual with a large amount of choice sets is expected to result in a lower response rate and/or lower response reliability [26]. To reduce the burden on respondents, a blocked design was used [15], which resulted in dividing the 48 choice sets of the efficient design into 3 questionnaire versions containing 16 choice sets each in which we ensured sufficient variation in attribute levels by finding blocks with near attribute level balance.

Each questionnaire started with the introduction of a hypothetical scenario (Figure S1). To facilitate comprehension of the DCE task, respondents were provided with detailed information about the attributes and attribute levels as well as with a clearly explained example of a choice task prior to preference elicitation. The main part of each questionnaire comprised 16 choice sets. In each choice set, respondents first received some additional information about the disease (i.e., the two scenario variables). Choice sets consisted of two unlabeled vaccination alternatives (vaccination A and vaccination B) and one opt-out alterative (see Figure S2 for a screenshot of a choice set). This opt-out was necessary since, as in real life, respondents are not obliged to take a vaccination. Respondents were asked to consider all three alternatives in a choice set as realistic alternatives and to choose the option that appealed most to them in the given situation.

Attributes needed to be described as clearly as possible in the choice sets since previous research has shown that respondents may have difficulties with interpreting probabilities [27] and that framing effects can influence DCE results [28]–[30]. Therefore, we included graphs to demonstrate percentages and rates, used realistic presentation of attributes (e.g. integers when discussing rates that included humans), and used cost as the last attribute. Furthermore, experts in the field of risk communication advised us on how to present the choice sets in this DCE. For example, we were advised to use the same type of graphs to present risks across both scenario variables and attributes. The last section of each questionnaire included questions on socio-demographic data and questions on previous experiences with vaccination. It also contained questions assessing experienced difficulty of the questionnaire (five-point scale). The questionnaire was presented to respondents in Dutch.

In order to test the survey, we conducted a formal pen and paper pilot with 29 respondents in the Netherlands. Additionally, we conducted five think-a-loud interviews [31] to qualitatively test for any problems in interpretation, for the understanding of the questions and to indicate whether respondents were providing a meaningful response. This resulted in minor changes to the layout and phrasing of the questionnaire. There was no need to adapt the selected combinations of scenarios or attribute levels of the DCE design. Since there were some adaptations to the questionnaire, data of these pre-tests were not included in the final analysis. The questionnaire is available from the authors on request.

To check the convergent validity of the DCE, we asked respondents to rank the five vaccination program attributes from most important to least important. External validation was not possible since we were using a hypothetical disease outbreak.

### Data collection

A market research company (Flycatcher) was hired to administer the online questionnaire to a representative sample of the general adult population of the Netherlands. Their online panel comprises 16,000 members and is ISO certified (ISO-26363). Recruitment of potential new members is done by digital media, paper invitations, face-to-face meetings and via intermediates. Assuming a participation rate of 50%, a random sub sample of 1,083 adult panel members (see sample size calculation below) was emailed a link to the questionnaire to participate in the current study. Quota sampling was used to ensure even distributions with respect to age, gender, education and region. A further quota was applied to each ‘questionnaire version’ to ensure comparable numbers of respondents in each of the three blocks of the design. Progress bars and error messages were incorporated to encourage completion. After completing the questionnaire, respondents were given the opportunity to comment on the questionnaire or topic at hand by filling out the free text question. The questionnaire was online for twelve days in June 2013, when the target number of 500 respondents was reached. All panel members gave informed consent prior to participating in the study and received a small incentive (€2.20, in the form of credits) for completion of the questionnaire.

### Sample size calculation

The mean sample size for DCE studies in health care published between 2005 and 2008 was 259, with nearly 40% of the sample sizes in the range of 100 to 300 respondents [32]. No adequate statistical methods exist to determine sample sizes for DCEs. Therefore, the rules of thumb as suggested by Orme [33] are frequently used. These rules recommend sample sizes for DCEs to be at least 300 respondents and suggest that also the number of tasks and alternatives should be taken into account when determining sample sizes. Based on this information, we aimed to have at least 500 respondents completing the questionnaire.

### Statistical analysis

To assess preference heterogeneity, we used a latent class model to analyze the DCE data. A latent class model [34], [35] can be used to identify the existence and the number of segments or classes in the population (i.e. identifying different utility (preference) functions across unobserved subgroups). Class membership is latent (i.e., unobserved) because each respondent belongs to each class up to a modeled probability and not deterministically assigned by the analyst a priori. The model is flexible in that the probability that sampled respondents belong to a particular class can be linked to covariates (e.g. age, gender), hence allowing for some understanding as to the make-up of the various class segments [34].

To account for the panel nature of the data, with each respondent completing 16 choice tasks, we used a panel version of the latent class model. In order to determine the number of classes, we selected the model with the best fit. We tested a number of different specifications for the utility function (e.g., categorical or numerical attribute levels, linearity, two-way interactions between all attributes and several attribute transformations, see Figure S3 for specifications of the functions) and selected the model with the lowest Akaike Information Criterion (AIC).

The latent class model estimates parameters in a class assignment model (which includes socio-demographic variables and thereby expresses the likelihood of a certain individual belonging to a certain class) and class-specific coefficients for each attribute (or interaction of attributes and scenario variables) in the utility function. For the class-specific coefficients and interactions, the statistical significance of a coefficient (P-value≤0.05) indicated that, conditional on belonging to that class, respondents considered the attribute important when making stated choices. In terms of the class assignment parameters, statistically significant parameter estimates indicate that the covariate can be used to distinguish between the different classes. For example, if the covariate male gender is negatively and significantly associated with a particular class in the assignment model, then this is indicative that men are less likely to belong to that particular class than women.

The sign of the coefficient reflects whether the attribute had a positive or negative effect on utility. The value of each coefficient represents the importance respondents assign to an attribute (level). However, different attributes utilize different units of measurement. For example the coefficient ‘effectiveness of the vaccine’ represented the importance per 10% protection rate. When looking at a vaccine that generates a 90% protection rate, the coefficient needs to be multiplied 9 times (9 times coefficient of ‘effectiveness of the vaccine’ of 10% = coefficient of ‘effectiveness of the vaccine’ of 90%).

We calculated class specific importance scores (IS) to visualize the relative importance of a given attribute in that class by dividing the difference in utility between highest and lowest level for a single attribute by the sum of the differences of all attributes for that class, taking interaction effects into account [36]. An attribute with an IS of 1 represents the most important attribute, while an attribute with an IS of 5 represents the least important attribute. Furthermore, we also calculated overall importance scores, by taking class probability into account.

### Expected uptake of the vaccine

Choice probabilities (mean uptakes) were calculated to provide a way to convey DCE results to policy makers that are more easily understandable. We calculated the choice probability (i.e. the mean uptake) for a base case vaccination for three given outbreaks by taking the exponent of the total utility for vaccination divided by the exponent of utility of both vaccination and no vaccination taking the class probabilities into account. The base-case vaccination program was chosen to resemble real life situations, and included the following attribute levels: vaccine effectiveness 70%, supposed to be a safe vaccine, advised by friends and positive traditional media attention, and no out-of-pocket costs. Outbreaks were defined as mild, moderate and severe (respectively a susceptibility and severity of 5% and 5%; 10% and 25%; and 20% 75%).

### Trade-offs

We calculated willingness-to-pay (WTP) values for the effectiveness of the vaccine attribute for mild, moderate and severe outbreaks (respectively a susceptibility and severity of 5% and 5%; 10% and 25%; and 20% and 75%). A WTP value represents how much one is willing to pay for a one unit change in the attribute of interest, and is calculated by taking the ratio of the derivative of the effectiveness attribute and the derivative of out-of-pocket costs. Since effectiveness was included as both a main effect and as part of an interaction effect with susceptibility to the disease and severity of the disease, it is necessary to calculate the derivatives with respect to all parts of the utility function where the attribute appears [37]. Because a latent class model was used, overall WTP measures can be calculated by weighing the conditional WTP values by the probability that respondents belong to a given class. We computed the confidence intervals using the Krinsky and Robb procedure [38] (Figure S3).

We used NLogit 4.0 software (www.limdep.com) to estimate the latent class models and SPSS 21.0 software (http://www-01.ibm.com/software/analytics/spss/) for all other analysis.

## Results

### Respondents

**Figure 1:**
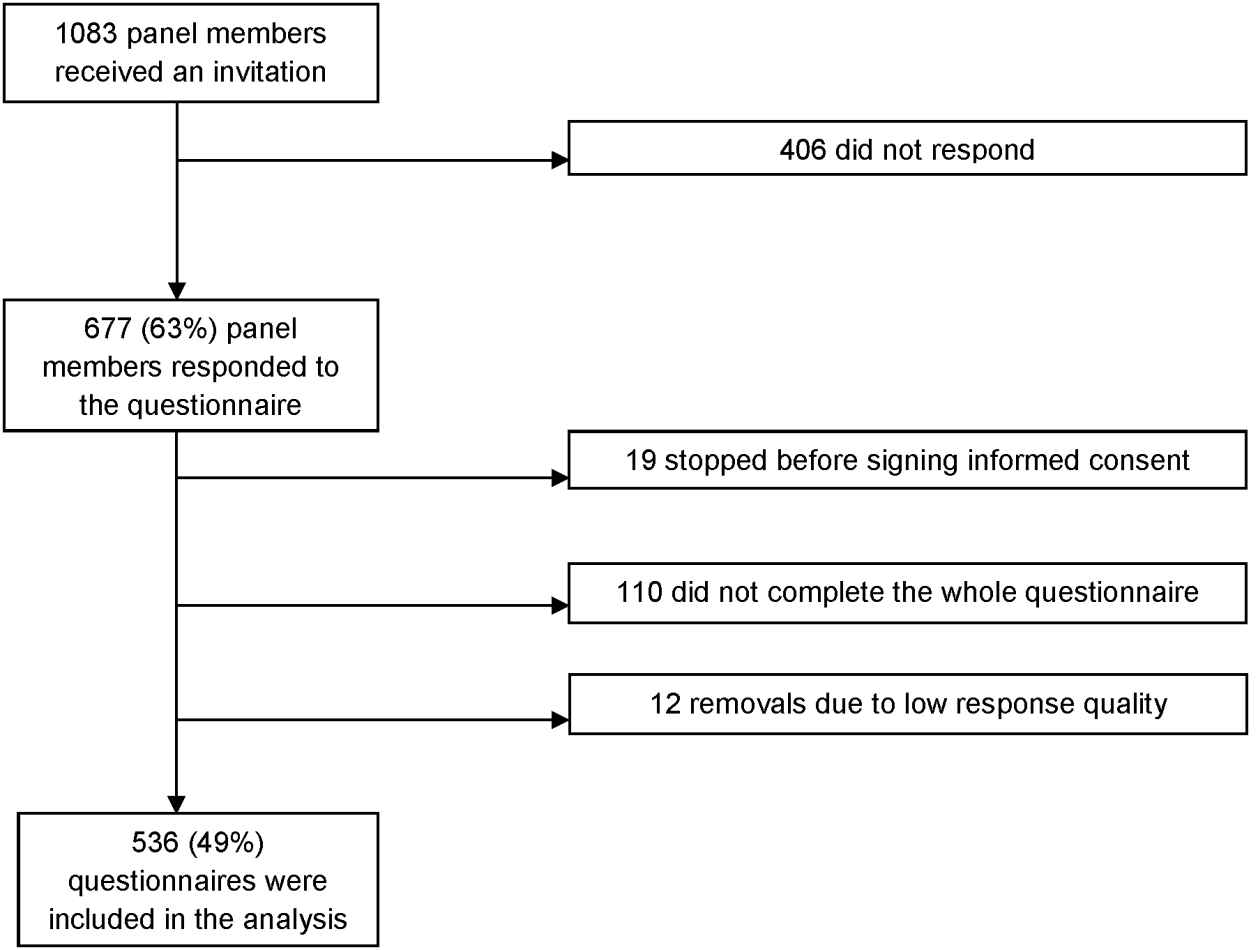
Overview of respondents accessing the study.

The participation rate was 677/1083 (63%, Figure 1), which reflected the expected response rate for this online panel. Of the 677 respondents, 548 completed the questionnaire. Respondents who completed the questionnaire did not differ regarding sex (p = 0.11) or educational level (p = 0.11) compared to respondents who did not complete the questionnaire. However, respondents who completed the questionnaire were younger (median age 50 vs. 53, p<0.01). Twelve respondents were excluded from the analysis, because they completed the questionnaire too quick; they completed the whole questionnaire in less than five minutes. Data of 536 (49%) respondents were included in the analysis.

Respondents had a median age of 50 years (interquartile range (IQR): 35–64), with a minimum of 18 and a maximum age of 89 years old (Table 2). 30% had a high educational level and 22% of the respondents indicated that they had a positive attitude regarding vaccination, i.e. that they would always get vaccinated. The sample was representative for the Dutch population regarding age, gender, educational level and region.

**Table 2:** Characteristics of respondents who completed the DCE survey (N = 536).

Characteristics	Subcategory	Sample statistics		CBS statistics
		*Median*	*IQR*	
Age in years		50	35–64	
		*n*	*%*	*%*
Age groups	18–24 years	49	9.2	11
	25–34 years	78	15	16
	35–44 years	84	16	19
	45–54 years	107	20	19
	55–64 years	92	17	16
	>65 years	126	24	19
Gender	Male	289	54	49
Country of birth	Netherlands	517	96	-
Educational level	Low	184	34	34
	Average	192	36	40
	High	160	30	26
Civil status	Married	296	55	-
	Registered partnership	48	9.0	-
	Unmarried	133	25	-
	Divorced	38	7.1	-
	Widow/widower	21	3.9	-
Children	Yes	345	64	-
Income in euros per year	Minimal (<11.000)	37	6.9	-
	Less than modal (11.000–23.000)	69	13	-
	Modal (23.000–34.000)	127	24	-
	1–2 times modal (34.000–56.000)	103	19	-
	2 times modal or more (>56.000)	78	15	-
	Do not know/do not want to say	122	23	-
Religion	Yes	244	46	-
Perception of health	Lower health than average	41	7.6	-
	Medium health	195	36	-
	Better health than average	300	56	-
Attitude regarding vaccination	Always get vaccinated	120	22	-
	Only if benefits > harms	259	48	-
	Only if benefits > harms, but I do not thinkthis is the case in the real world	116	22	-
	Never get vaccinated, even ifbenefits > harms	41	7.6	-
Belongs to target group for seasonal flu vaccine	Yes	239	45	-
	No	270	45	-
	No, but receives flu vaccination via work	27	5.0	-
Belongs to the target group and received seasonal fluvaccination last year	Yes	160	60	-

Note: Abbreviations used: CBS = Statistics Netherlands, IQR = Interquartile range.

The median completion time of the whole questionnaire was 13 minutes (median, IQR: 9.6–19). It took respondents a median of 6.3 minutes (IQR: 4.2–9.6) to complete 16 choice tasks. The time respondents needed to fill in one choice task decreased from a median of 39 seconds (IQR: 20–62) for choice task 1 to 15 seconds (IQR: 10–24) for choice task 16. 67% of the respondents marked the number of choice tasks as ‘exactly the good number’ and 76% marked the questions as clear or very clear. A minority (13%) of the respondents found the questions hard or very hard to answer. Most of the respondents found the topic interesting or very interesting (87%). Responses to the free text question indicated that respondents felt that they were adequately informed to answer the questions in the questionnaire.

**Figure 2:**
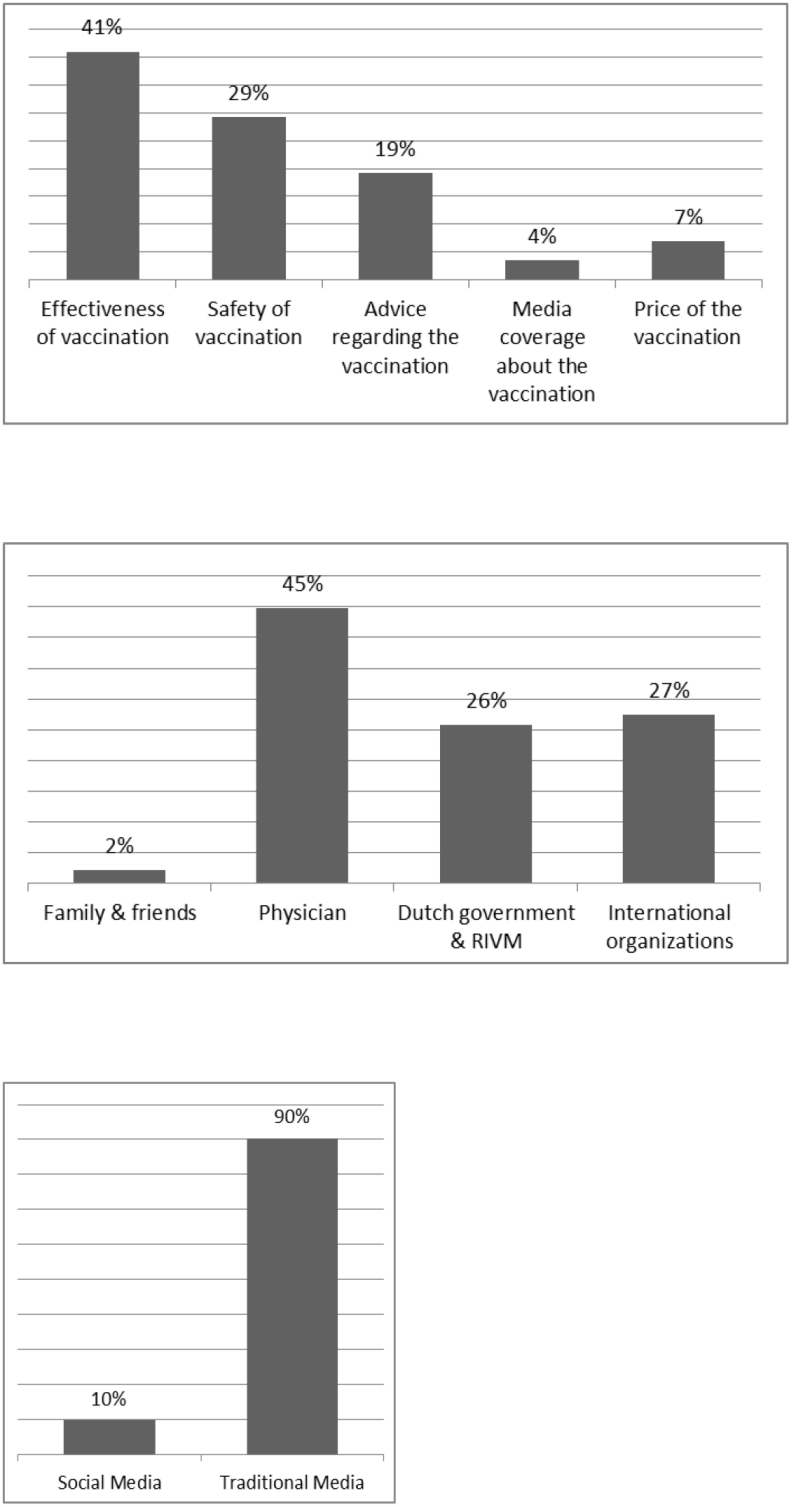
**a. Direct ranking of attributes.** Note: The percentages represent the proportion of people that ranked that vaccination program attribute as most important when deciding on vaccination. **b. Direct ranking of attribute levels.** Note: The percentages represent the proportion of people that ranked that vaccination program attribute level of advice regarding the vaccination as most important when deciding on vaccination. **c. Direct ranking of attributes levels.** Note: The percentages represent the proportion of people that ranked that vaccination program attribute level of media coverage about the vaccination as most important when deciding on vaccination.

Direct ranking showed that respondents considered effectiveness the most important vaccine specific attribute, followed by safety of the vaccine and advice regarding the vaccine (Figure 2a–2c). Respondents marked their doctors’ advice as most important, followed by the advice of international organizations and the advice of the Dutch government & National Institute of Public Health and the Environment (Dutch abbreviation: RIVM). Traditional media influenced the decision regarding vaccination more than social media.

### Discrete choice experiment results

The ‘no vaccination’ option was chosen in 37% of the choice sets. 61 respondents (11.0%) always chose the ‘no vaccination’ option. 113 respondents (21%) never chose the ‘no vaccination’ option.

Using a latent class model, two classes were identified (Table 3). The average class probabilities within the sample were 0.63 for class 1 and 0.37 for class 2. The probability to belong to a specific class was dependent on two socio-demographic variables: the sex of the respondent and the attitude of the respondent regarding vaccination. Males and individuals who stated that they (possibly) wanted to get vaccinated had the highest chance to belong to latent class 1, while females and individuals who stated that they would never get vaccinated had the highest chance to belong to latent class 2. Other socio-demographic variables were not significantly explaining class assignment probabilities.

**Table 3:** Preferences of respondents for vaccinations in pandemic situations based on a panel latent class logit model (N = 536).

Attributes		Latent class 1					Latent class 2			Overall
		*Value^2^*		*S.E.*		*IS^3^*	*Value^2^*	*S.E.*	*IS^3^*	*IS^3^*
Constant (no vaccination)		0.22**		0.05			2.46**	0.15		
Effectiveness of vaccine (per 10%)		0.07**		0.01		1	0.01	0.03	1	1
Side effects unknown, but expected to be safe (reference)^1^		0.21		-		4	0.27	-	5	5
Side effects unknown, no experience yet		−0.21**		0.01			−0.27**	0.05		
Family and/or friends recommend (reference)^1^		−0.33		-		2	−0.04	-	3	2
Family and/or friends discourage		−0.50**		0.03			−0.39**	0.11		
Your doctor recommends		0.42**		0.03			0.51**	0.10		
Your doctor discourages		−0.67**		0.03			−1.02**	0.15		
Dutch government & RIVM recommend		0.58**		0.03			0.48**	0.10		
International organizations recommend		0.49**		0.03			0.45**	0.09		
Traditional media is positive (reference)^1^		0.15		-		5	0.39	-	4	4
Traditional media is negative		−0.20**		0.03			−0.28**	0.09		
Social/interactive media is positive		0.16**		0.02			0.18*	0.08		
Social/interactive media is negative		−0.11**		0.03			−0.29**	0.09		
Out-of-pocket costs of the vaccination (per 10 euro)		−0.06**		0.00		3	−0.17**	0.01	2	3
Interaction: effectiveness of vaccine (per 10%)×susceptibility to the disease (per 10%)		0.12**		0.00		-	0.11**	0.00	-	-
Interaction: effectiveness of vaccine (per 10%)×severity of the disease (per 10%)		0.02**		0.00		-	0.01**	0.00	-	-
**Class probability model**	**Subcategory**									
Constant		-	-		-		1.63**	0.47	-	-
Sex	Male	-	-		-		−0.80**	0.22	-	-
Attitude regarding vaccination	I will never get vaccinated (reference level)	-	-		-		<0.01	<0.01	-	-
	I will always get vaccinated	-	-		-		−4.09**	0.73	-	-
	I will only get vaccinated if advantages>disadvantages	-	-		-		−1.88**	0.50	-	-
	I will only get vaccinated if advantages>disadvantages, however I do not thinkthat is the case in the real world	-	-		-		−0.78	0.48	-	-
**Class probability**										
Average		0.63	-		-		0.37	-	-	-
**Model fit**										
AIC		1.64	-		-		-	-	-	-
Log likelihood		−6989	-		-		-	-	-	-
R^2^		0.26	-		-		-	-	-	-

Notes: Effects coded variables used for safety of the vaccine, advice about the vaccine, media coverage about the vaccine. Number of observations: 25728 (16*3*536). Abbreviations used: S.E. = standard error. IS = importance score. RIVM = Dutch abbreviation for National Institute for Public Health and the Environment.

(1) The values of the vaccination program attributes reference levels equals the negative sum of the coefficients of the included attribute. (2) **, * denotes significance at the 1% and 5% respectively. (3) The IS were calculated for a severe outbreak with a susceptibility to the disease of 20% and a severity of the disease of 75%.

The sign of the coefficient indicates whether the attribute had a positive or negative effect on utility (Table 3). For example, the positive sign for effectiveness and for side effects unknown, but expected to be safe indicated that an effective and safe vaccination was preferred over a vaccination which was less effective and with which there was no experience yet. The negative sign for out-of-pocket costs of vaccination indicated that respondents preferred vaccinations with lower out-of-pocket costs. The positive sign of the constant indicates that, everything else being equal, respondents preferred no vaccination over vaccination.

Nearly all of the vaccine specific characteristics were statistically significant (Table 3), proving to influence respondents’ preference for vaccination. The interactions between the disease specific characteristics and effectiveness were significant and positive. This indicates that the preference for the level of effectiveness of a vaccination is dependent upon the levels of severity and susceptibility. If the susceptibility to or severity of a disease are higher, while the effectiveness of a vaccination is the same, preference for vaccination increases relative to no vaccination. In other words, if the susceptibility to the disease or the severity of a disease is higher, lower vaccination effectiveness will result in the same utility level. Note that the two disease characteristics cannot be included as a main effect but only as interaction effects, since they are scenario variables that are constant across all vaccine alternatives. All other 2-way interactions were not statistically significant.

When comparing the overall importance scores (Table 3) with the direct ranking question (Figure 2a), effectiveness of the vaccine was considered the most important attribute in both preference elicitation methods, especially when an outbreak was more serious, and media coverage of the vaccine as the least important attribute. These results support the convergent validity of the results.

Preference heterogeneity was substantial; respondents belonging to latent class 1 seemed to place more weight on the effectiveness of the vaccine than respondents of latent class 2 (IS of 2 for class 1 compared to an IS of 5 for class 2, in case of a mild outbreak). However, in case of a severe outbreak, effectiveness was the most important attribute for both latent classes. Respondents belonging to class 2 were more influenced by the media and more sensitive to costs than respondents belonging to latent class 1 (respectively an IS of 3 and 1 for class 2, and an IS of 5 and 3 for class 1, in case of a mild outbreak of the disease). For respondents of both classes, the advice regarding vaccination of others was important. Respondents in class 1 were most influenced by the advice of the government & RIVM and international organizations, while respondents in latent class 2 were most influenced by the recommendation or discouraging of their physician.

### Trade-offs

Based on the expressed preferences, respondents were willing to pay €6.0 (95% Confidence Interval: €3.7–€8.3) to receive a 10% more effective vaccine in case of a mild pandemic outbreak (Table 4). If a pandemic outbreak was more severe the willingness to pay for a vaccine which was 10% more effective increased up to €20 (€18–€22) in case of a moderate outbreak and €39 (€36–€44) in case of a severe outbreak.

**Table 4:** Willingness to pay.

Attribute	To receive a vaccination	WTP (€, CI)		
		Mild pandemic^1^	Moderate pandemic^2^	Severe Pandemic^3^
Effectiveness of vaccine	With 10% more effectiveness	6.0 (3.7–8.3)	20 (18–22)	39 (36–44)

Notes: Abbreviations: WTP = willingness to pay; € = euro; CI = 95% confidence interval based on the Krinsky and Robb method adjusted for class probabilities and taking into account interaction effects (see Figure S3 for more information). (1) Mild pandemic is defined as a disease with a susceptibility of 5% and a severity of 5%. (2) Moderate pandemic is defined as a disease with a susceptibility of 10% and a severity of 25%. (3) Severe pandemic is defined as a disease with a susceptibility of 20% and a severity of 75%.

### Expected uptake of the vaccine

**Figure 3:**
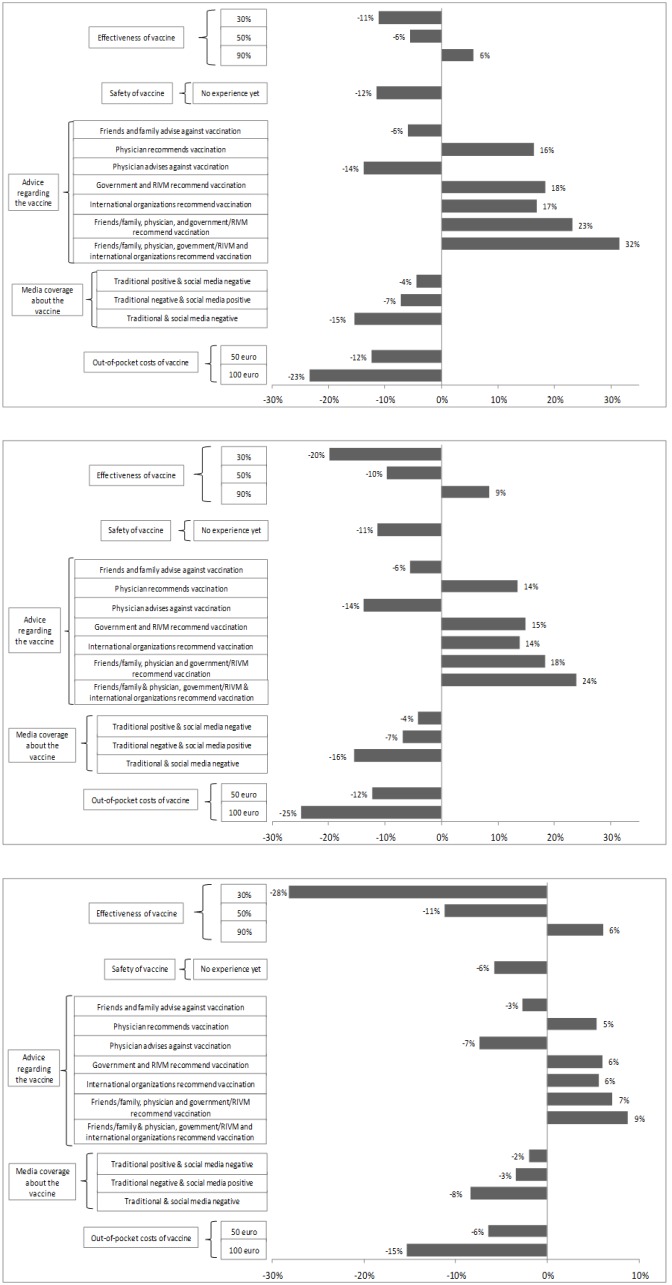
**a. Estimates for predicted probability of participation; values for a mild outbreak.** Notes: (1) The percentages represent the change in probability compared to base case vaccination. (2) The base case vaccination is 70% effective, supposed to be safe, recommended by friends/family, the traditional media is positive and there are no out-of-pocket costs. This base case is indicated as zero change in the probability of the x-axis. (3) A mild outbreak is defined as 5% of the population getting sick and 5% of the population getting severe symptoms. (4) Probability of base case vaccination in this scenario = 50%. **b. Estimates for predicted probability of participation; values for a moderate outbreak.** Notes: (1) The percentages represent the change in probability compared to base case vaccination. (2) The base case vaccination is 70% effective, supposed to be safe, recommended by friends/family, the traditional media is positive and there are no out-of-pocket costs. This base case is indicated as zero change in the probability of the x-axis. (3) A moderate outbreak is defined as 10% of the population getting sick and 25% of the population getting severe symptoms. (4) Probability of base case vaccination in this scenario = 65%. **c. Estimates for predicted probability of participation; values for a severe outbreak.** Notes: (1) The percentages represent the change in probability compared to base case vaccination. (2) The base case vaccination is 70% effective, supposed to be safe, recommended by friends/family, the traditional media is positive and there are no out-of-pocket costs. This base case is indicated as zero change in the probability of the x-axis. (3) A severe outbreak is defined as 20% of the population getting sick and 75% of the population getting severe symptoms. (4) Probability of base case vaccination in this scenario = 88%.

The mean predicted uptake of the base-case vaccination program increased from 50% in a mild pandemic up to 88% for a severe pandemic (Figure 3a–3c). The more serious an outbreak was, the more the predicted uptake depended on effectiveness of a vaccine, e.g. a vaccine that was 40% less effective compared to the base case vaccination decreased the vaccination uptake 11, 20 and 28 percent points, for a mild, moderate or severe outbreak respectively. Irrespective of the disease scenario; higher out-of-pocket costs had a relatively large impact on the vaccination uptake, compared to the base case vaccination which was free. Furthermore, recommendation of the vaccine by physicians, the government & RIVM or international organizations resulted in a substantial increase of the predicted uptake of the base case program (e.g. an increase of 16, 18 and 17 percent points respectively in case of a mild outbreak). Assuming that all bodies advised positively regarding the vaccine (including friends and family) the predicted uptake increased with 32 percent points in case of a mild outbreak.

## Discussion

This DCE showed that effectiveness, safety and out-of-pocket costs of the vaccine, as well as advice regarding and media coverage about the vaccine all influenced the general populations’ preference for pandemic vaccinations. Preference heterogeneity was substantial; two latent classes with different preferences were identified by a latent class model. Female respondents and individuals who stated that they would never get vaccinated were more influenced by the media and more sensitive to costs than male respondents and individuals who stated that they were (possibly) willing to get vaccinated. As expected, respondents preferred and were willing to pay more for more effective vaccines, especially if the outbreak was more serious. Changes in effectiveness, out-of-pocket costs of the vaccine and in the body that advises the vaccine substantially influenced the predicted uptake.

This is the first DCE investigating how characteristics of pandemic vaccinations influence preferences for vaccination programs in different pandemic outbreaks. Two systematic reviews assessed which factors were associated with uptake of the Influenza A (H1N1) pandemic vaccine. These also showed the need for targeted messaging to reach vaccination goals [7], [8]. Especially the conclusion of one of these reviews [7] that social pressure and confidence in sources of information had an effect on the intention to vaccinate, is in line with our results. To gain insight in factors explaining willingness to vaccinate against Influenza A(H1N1) in The Netherlands, a questionnaire study was conducted among the general Dutch population during the 2009–2010 pandemic [39]. Similar results as we found were reported: people who were afraid of the disease, who perceived it as a severe disease, who believed in the efficacy of the vaccine and who trusted the information the government provided had higher odds for vaccination. Furthermore, the majority of respondents trusted the information provided by their general practitioner and more than half of the respondents trusted the information provided by the Dutch government and RIVM. Another questionnaire study regarding the Influenza A(H1N1) pandemic in the Netherlands showed results that are in line with our results as well; most respondents wanted to receive information about infection prevention from municipal health services, health care providers, and the media. Higher levels of intention to receive vaccination were associated with increased government trust, fear or worry about the disease, and perceived vulnerability to the disease [40]. Several DCEs on non-pandemic vaccines [12], [13], [41] showed the influence of similar characteristics on vaccination preferences as we found in our study. In a DCE on preferences for HPV vaccination [12], it was found that the degree of protection positively influenced the preference of girls for vaccination, while the risk of side effects had a negative effect. A DCE among parents preferences for influenza vaccination for their children [13], showed that the efficacy of a vaccination and the recommendation of physicians positively influenced parents’ preferences, while the risk of temporary side effects had a negative effect. A DCE on marginal WTP for HIV vaccines [41] found that biomedical characteristics of a hypothetical HIV vaccine, such as efficacy, vaccine induced seropositivity and side effects, were the most important attributes for vaccination programs.

Our results suggest that side effects of the vaccine are less important than the other included attributes when deciding on vaccinations, while in other studies (including DCEs) safety of vaccinations was dominant [7], [12], [13], [41], [42]. This difference can probably be assigned to the choice of attribute levels since respondents in the current DCE were informed that the chance of side effects was expected to be low and either comparable to vaccines that are already on the market or expected to be low, but with no experience with a similar vaccination yet, i.e. a totally new vaccination. Our study showed that preference heterogeneity was substantial. Findings on heterogeneity are supported by a focus group study on acceptance of hypothetical pandemic vaccinations in Canada, where parents with non-mainstream beliefs showed different concerns regarding vaccinations [43].

This study had several limitations. First, we measured preferences for hypothetical vaccines in hypothetical pandemic outbreaks. Although we were not able to measure the external validity of our results, the results may be very helpful in helping to prepare for pandemic outbreaks. Additionally, the signs of the coefficients were generally consistent with our a priori hypothesis (a higher susceptibility to the disease, a higher severity of the disease and a higher effectiveness would have a positive effect on vaccination) and therefore, theoretically valid. Second, the participation rate of 49% was not optimal and selection bias cannot be excluded. However, this participation rate is equal or even higher than most other DCEs in health care. Furthermore, the participation rate was comparable to the average rate of the internet panel we used. We expect our results to be generalizable since age, gender, level of education and region of our sample are comparable to that of the general population of the Netherlands. Third, due to both the number and the type of attributes and levels that respondents needed to take into account when completing the choice tasks, it can be expected that respondents might have experienced difficulties, which might have influenced the results. However, piloting and think-a-loud interviews in the preparation phase, as well as questions that assessed the experienced difficulty of the questionnaire showed that the majority of respondents had no problems with completing the tasks. Fourth, we included safety of the vaccine as a categorical attribute, instead of a numerical attribute, which would have helped respondents to compare risks of vaccinations with risks of the disease. However, when designing the DCE, expert interviews showed that safety of the vaccine is more or less a fixed attribute (either being sure that the vaccine will be safe, or that there is no experience with the vaccine yet and there is thus a chance of long-term side effects). Therefore, we included the safety of the vaccine as an attribute with categorical levels.

Insights in the factors influencing the intention to accept or decline a pandemic vaccine may have implications for both national and international policy and for further research. When communicating public health messages regarding vaccination, one should be aware of preference heterogeneity and therefore use different sources and channels to distribute the messages [44]. The current study provides guidance on how to target public health messages, by the identification of two classes with different preferences for pandemic vaccinations. To immediately reduce the number of susceptible people, a possible strategy could be to target the message for the first phase of a vaccination program to the more vaccination minded persons, here latent class 1. This can be done by using the government and RIVM as bodies to advice the vaccine to males and focus more on the expected effectiveness of the vaccine. Next, physicians can advise females to take the vaccine. Additionally, out-of-pocket costs need to be as low as possible, as our study showed the negative relation between out-of-pocket costs and vaccination decisions. For public health messages during vaccination programs, it is also important to monitor side effects. Updates of the side effects of the vaccine need to be given on a regularly basis to make sure that an informed choice can be made and to reduce fear of the side effects of the vaccine. Furthermore, policy makers can use the expected uptake probability of hypothetical vaccinations when predicting the number of vaccinations that is needed. Although these numbers are rough estimates and it is not known if they are externally valid, the expected uptake can still be useful when other information is lacking. Additionally, these numbers can guide communication on the expected vaccination uptake. Since this is the first quantitative study in motivations for pandemic vaccinations, we do not know to what extent differences exist between countries regarding preferences for vaccinations. There is some evidence, including a questionnaire study in four countries investigating reasons why high risk people reject influenza vaccination in four countries of Europe suggests differences between respondents of the different countries [45]. Therefore, further international research is recommended.

We conclude that various disease and vaccination program attributes influence respondents’ preferences for pandemic vaccination programs. Agencies responsible for preventive measures during pandemics can use the findings of this study that out-of-pocket costs and the way advice is given affect vaccination uptake to change the way vaccination is marketed during future pandemic outbreaks. The preference heterogeneity shows that information regarding vaccination needs to be targeted differently depending on gender and willingness to get vaccinated.

## Supporting Information

Figure S1 **Hypothetical scenario.**
(DOCX)

Figure S2 **Example of a choice set.**
(DOCX)

Figure S3 **Utility functions.**
(DOCX)
