# Supplementary material for: Acceptance of Vaccinations in Pandemic Outbreaks: A Discrete Choice Experiment
Source: PLoS One. 2014 Jul 24;9(7):e102505. doi: 10.1371/journal.pone.0102505 (PMC4109921; doi:10.1371/journal.pone.0102505)
Supplement: Figure S1 — Hypothetical scenario. (DOCX) [file pone.0102505.s001.docx]

**Supporting Information 1: Hypothetical scenario.**

*This scenario was presented to respondents in Dutch.*

“Imagine, a new disease emerged abroad. This disease is highly contagious, because the disease spreads by droplets. All over the world, people become infected, also in the Netherlands. You can do some things yourself to prevent you of getting the disease (such as washing hands, etc.). However, the most effective preventive measure is a vaccination. Vaccinations are available for everyone in the Netherlands”
