# Supplementary material for: Acceptance of Vaccinations in Pandemic Outbreaks: A Discrete Choice Experiment
Source: PLoS One. 2014 Jul 24;9(7):e102505. doi: 10.1371/journal.pone.0102505 (PMC4109921; doi:10.1371/journal.pone.0102505)
Supplement: Figure S2 — Example of a choice set. (DOCX) [file pone.0102505.s002.docx]

**Supporting Information 2: Example of a choice set.**

*Choice sets were presented to respondents in Dutch.*

Additional information regarding the new disease (the situation without vaccination):

| **Susceptibility to the disease** | **50 out of 1.000** people will get sick (develop symptoms) |
| --- | --- |
| **Severity of the disease** | **25% of all sick people** will get severe symptoms |

You can choose the following 3 options, what do you choose?

|  | **Option 1:**  **No vaccination** | **Option 2:**  **Vaccination A** | **Option 3:**  **Vaccination B** |
| --- | --- | --- | --- |
| **Effectiveness of the vaccine** | **n.a.**  This means:  **50 out of 1.000**  people will get sick  **25% of these sick people**  will get severe symptoms | Effectiveness: **30%**    This means:  **15 out of 1.000**  people will not get sick,  due to vaccination  **35 out of 1.000**  people will get sick,  despite the vaccination  **25% of these sick people**  will get severe symptoms | Effectiveness: **70%**  This means:  **35 out of 1.000**  people will not get sick,  due to vaccination  **15 out of 1.000**  people will get sick,  despite the vaccination  **25% of these sick people**  will get severe symptoms |
| **Safety of the vaccine**  **(long term severe side effects)** | No side effects | Unknown, but  expected to be safe | Unknown,  no experience yet |
| **Advice about the vaccine** | n.a. | Recommended by  your doctor | Recommended by family  and friends |
| **Media coverage about the vaccine** | n.a. | Radio, newspapers  and television positive | Radio, newspapers  and television negative |
| **Out-of-pocket costs** | 0 euro | 50 euros | 50 euros |

| **What do you choose for yourself?**  (please thick one box only) |  |  |  |
| --- | --- | --- | --- |
