# Supplementary material for: Acceptance of Vaccinations in Pandemic Outbreaks: A Discrete Choice Experiment
Source: PLoS One. 2014 Jul 24;9(7):e102505. doi: 10.1371/journal.pone.0102505 (PMC4109921; doi:10.1371/journal.pone.0102505)
Supplement: Figure S3 — Utility functions. (DOCX) [file pone.0102505.s003.docx]

**Supporting Information 3: Utility functions.**

The utility function for no vaccination in the model with the best fit was:

V (no vaccination) _nsj|c_ = β _0|c_ (Eq.1)

The utility function for vaccination in the model with the best fit was:

V (vaccination) _nsj|c_ = β _1|c_ effectiveness _nsj|c_ + β _2|c_ side effects (unknown) _nsj|c_ + β _3|c_ advice (friends advise against) _nsj|c_ + β _4|c_ advice (doctor recommends) _nsj|c_ + β _5|c_ advice (doctor advises against)_nsj|c_ + β _6|c_ advice (government & RIVM recommend) _nsj|c_ + β _7|c_ advice (international organizations recommend) _nsj|c_ + β _81|c_ media coverage (traditional media negative) _nsj|c_ + β _9|c_ media coverage (social media positive) _nsj|c_ + β _10|c_ media coverage (social media negative) _nsj|c_ + β _11|c_ costs _nsj|c_ + β _12|c_ effectiveness _nsj|c_ x susceptibility _nsj|c_ + β _13|c_  effectiveness _nsj|c_ x severity _nsj|c_ (Eq.2)

where

V_nsj|c_ represents the observable utility that respondent *n* belonging to class segment *c* has for alternative *j* in choice set s for vaccination;

β _0|c_ is an alternative-specific constant reflecting respondents’ preferences for no vaccination compared to receive a vaccination for a certain class;

β _1-11|c_ are class-specific main-effects regression coefficients of the attributes, indicating the relative weight individuals place on certain attribute levels.

β _12-13|c_ are class-specific two-way interaction effects (i.e. an effect where the influence of one attribute depends on the level of another attribute).

In addition to the utility function for vaccination (Eq. 1 and Eq. 2), the final model allowed for several covariates to enter into the class assignment model. The class assignment utility function for the final model was:

V_nc_ = β _0|c_ + β _1|c_ gender _n_ + β _2|c_ attitude to vaccination (always positive) _n_ + β _3|c_ attitude to vaccination (in favor when advantages > disadvantages) _n_ + β _4|c_ attitude to vaccination (in favor when advantages > disadvantages, but don’t thinks that’s the case in the real world) _n_  (Eq. 3)

The WTP was calculated by taking the ratio of the parameter for the effectiveness attribute to the parameter related to out-of-pocket costs. Since effectiveness was included as both a main and an interaction effect, it was necessary to take also values of the attributes susceptibility to the disease and severity of the disease into account when calculating the WTP (de Bekker-Grob EW, Rose JM, Bliemer MC (2013) A Closer Look at Decision and Analyst Error by Including Nonlinearities in Discrete Choice Models: Implications on Willingness-to-Pay Estimates Derived from Discrete Choice Data in Healthcare. Pharmacoeconomics 31: 1169-1183). Because a latent class model was used, overall WTP measures could be calculated by weighing the conditional WTP values by the probability that respondents belong to a given class (given by the class assignment probability in Equation (1) and (2)). We calculated the WTP for three different pandemic outbreaks, using the values 5%, 10% and 20% for susceptibility and the values 5%, 25% and 75% for severity.
